# Can Short-Term Use of Electronic Patient Adherence Monitoring Devices Improve Adherence in Patients Failing Second-Line Antiretroviral Therapy? Evidence from a Pilot Study in Johannesburg, South Africa

**DOI:** 10.1007/s10461-016-1417-7

**Published:** 2016-05-04

**Authors:** Denise Evans, Rebecca Berhanu, Faith Moyo, Arthemon Nguweneza, Lawrence Long, Matthew P. Fox

**Affiliations:** 1 Health Economics and Epidemiology Research Office, Department of Internal Medicine, School of Clinical Medicine, Faculty of Health Sciences, University of the Witwatersrand, Johannesburg, South Africa; 2 Right To Care, Johannesburg, South Africa; 3 Department of Global Health, Boston University School of Public Health, Boston, MA USA; 4 Department of Epidemiology, Boston University School of Public Health, Boston, MA USA

**Keywords:** Second-line, Electronic adherence monitoring device (EAMD), Wisepill™, Adherence, Viral suppression

## Abstract

High levels of adherence are required to achieve the full benefit of ART. We assess the effectiveness of electronic adherence monitoring devices among patients failing second-line ART, as measured by viral load suppression. Cohort study of Wisepill™ real-time adherence monitoring in addition to intensified adherence counselling over 3 months in adults with a viral load ≥400 copies/ml on second-line ART in Johannesburg, South Africa between August 2013 and January 2014. Patients were sent SMS reminders upon missing a scheduled dose. We compared outcomes to earlier historical cohorts receiving either intensified adherence counselling or adherence counselling alone. Overall, 63 % of the participants (31/49) took >80 % of their prescribed medication; this dropped from 76 to 53 and 49 % at 1, 2 and 3 months post-enrolment respectively. Compared to those with good adherence (>80 %), participants with poor adherence (≤80 %) had a higher risk for a subsequently elevated viral load ≥400 copies/ml (relative risk (RR) 1.47 95 % CI 0.97–2.23). Participants found the intervention “acceptable and useful” but by 6 months after eligibility they were only slightly more likely to be alive, in care and virally suppressed compared to those who received intensified adherence counselling (44.9 vs. 38.5 %; RR 1.19; 95 % CI 0.85–1.67) or adherence counselling alone (44.9 vs. 40.9 %; RR 1.12; 95 % CI 0.81–1.56). In patients with an elevated viral load on second-line ART electronic adherence monitoring was associated with a modest, but not significant, improvement in viral suppression.

## Introduction

The advent of antiretroviral therapy (ART) has led to significant reductions in morbidity and mortality [1–3]. Despite the huge successes in increasing HIV treatment coverage, most patients who experience virologic failure on second-line ART in low-middle income countries fail due to poor adherence rather than resistance to a class of ART drugs [4, 5]. For patients on second-line protease inhibitor-based ART, high levels (≥80 %) of adherence are required for viral suppression and poorer outcomes are observed when adherence drops [6, 7].

Some patients demonstrating poor adherence go on to fail therapy, develop resistance and require more expensive subsequent treatment regimens [8–12]. In most resource-limited settings, access to second-line treatment is limited and access to third-line is non-existent [13]. In South Africa, second-line treatment is readily available but comes at a significantly higher cost compared to first-line therapy, which forces a shift of resources away from initiating new patients onto treatment [11]. Delaying the need for second- and third-line therapy through improved treatment adherence is of the utmost importance for maximizing existing resources. The cost of third-line regimens has been estimated to be more than 15 times that of first-line and six times that of second-line regimens.

While several interventions to improve adherence exist [14, 15], most can only identify poor adherence after it has already become a problem [16]. Some studies report that 50 % of non-adherent patients may experience loss of viral suppression after a 14 day lapse in adherence [17], therefore highlighting the need to identify poor adherence early. Real-time electronic adherence monitoring devices (EAMD) have been proposed for improving adherence as it presents the opportunity to identify missed doses in as little as 24–48 h. While these devices are often used in clinical trials of HIV drugs, recent studies have demonstrated that the data from these devices can be effectively used to improve patient adherence or reduce treatment interruptions of >72 h in the routine outpatient ART clinic setting [18, 19]. These real-time, wireless adherence monitoring strategies for ART may provide novel opportunities to proactively prevent virologic rebound and treatment failure [17]. While these devices have been shown to be effective in first-line patients [16, 17, 19] there are questions as to whether such a strategy is cost effective. To date, no study has focused on patients with an elevated viral load on second-line ART, a population that would be both at high risk for poor adherence and in whom preventing the need for very expensive and difficult to access third-line regimens could prove to be cost-effective.

We conducted a pilot study to assess the effectiveness of EAMD in achieving adherence among adult patients with an elevated viral load (≥400 copies/ml) on second-line ART. Adherence was defined as achieving a suppressed viral load (<400 copies/ml) between 3 and 6 months after an elevated viral load (≥400 copies/ml) on second-line ART. We compare outcomes to two historical cohorts receiving the standard of care; the one cohort received intensified adherence counselling while the other received standard adherence counselling. In addition to generating preliminary estimates of effectiveness, we generate preliminary cost data and evaluate the feasibility of EAMD.

## Methods

### Study Site

This study was conducted among HIV-positive adult (≥18 years) patients receiving treatment at the Themba Lethu Clinic in Johannesburg, South Africa. The Themba Lethu Clinic (TLC) cohort has been described elsewhere [20]. Themba Lethu is a large public sector HIV comprehensive care, management and treatment (CCMT) site that follows the national HIV treatment guidelines [21–23]. Patients who fail first-line therapy are switched to a regimen containing a protease inhibitor (PI), typically lopinavir-ritonavir (LPVr) and two nucleoside reverse transcriptase inhibitors (NRTI). HIV viral load testing is used in South Africa to determine when a patient is considered to have failed an ART regimen.

By January 2014, Themba Lethu had initiated over 22,000 adult patients onto ART and of these 2790 have initiated second-line therapy. Of the patients that have initiated second-line ART, close to 20 % received intensified adherence counselling for an elevated viral load while on second-line ART.

### Study Design and Population

**Fig. 1:**
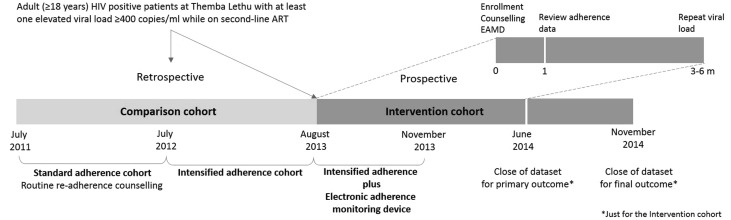
Schematic representation of the enrolment periods for the comparison and intervention cohorts. A summary of the visit schedule for the intervention cohort is provided in the *top right corner* of the schematic

We conducted an ambi-directional cohort study comparing patients in the intervention cohort (EAMD in addition to intensified adherence counselling) to two historical comparison cohorts. Eligible patients were adult (≥18 years) HIV positive patients at Themba Lethu who were receiving a second-line ART regimen containing lopinavir/ritonavir or atazanavir/ritonavir and experienced a single elevated viral load (≥400 copies/ml) on second-line ART (Fig. 1).

*Comparison cohorts*: Comparison cohorts for the study were chosen from an electronic patient medical record database at the clinic. In order to compare patients who received the intervention to patients who were managed using other approaches, we included two comparison cohorts from two different time periods prior to the use of Wisepill™ devices.

1. The first and historically earliest cohort called the “standard adherence cohort” included patients managed according to the standard of care at the clinic prior to the introduction of second-line clinic (between July 2011 and July 2012). These patients received routine re-adherence counselling with a counsellor or social worker and the viral load was repeated 3 months later. Genotyping and third-line drugs were not available during this period.
2. The second, called the “intensified adherence cohort” included patients enrolled in the second-line ART clinic between July 2012 and the start of the intervention period (August 2013). Since July 2012 Themba Lethu has operated a second-line ART clinic focused on managing patients who are at high risk of failing second-line therapy (i.e., have an elevated viral load ≥400 copies/ml while on second-line ART). Patients are typically identified using an electronic medical record called TherapyEdge-HIV™. Patients with high viral loads are flagged for enhanced adherence counselling at their next clinic visit (1–2 months). The clinic manages these patients together during normal clinic hours however these patients bypass normal queues and undergo a longer intensified adherence counselling session than normal with an experienced counsellor or social worker. Patients then meet with a senior clinician that same day to address any adherence problems identified during counselling and additional issues pertaining to treatment side effects. Unlike the general clinic, attempts are made for patients to be seen by the same clinician at subsequent visits to maintain continuity and establish a patient-provider relationship. The patient is advised to continue their current regimen and return 3 months later to retest whether their viral load is suppressed. Patients that re-suppress (<400 copies/ml) their viral load at this point return to the normal clinic flow whereas those that fail to re-suppress are either sent for HIV genotype testing if the clinician suspects drug resistance or re-adherence counselling if the clinician suspects that adherence has not improved. Both resistance testing and third-line drugs (etraverine, darunavir, raltegravir) were available at the site after 2012 through a PEPFAR grant.

*Intervention cohort*: In addition to intensified adherence counselling (which was the standard of care at the time), the intervention included the use of an EAMD until the first follow-up viral load test (3-6 months after enrollment). The intervention was designed to complement standard of care at the clinic, with little disruption to clinic duties or flow and apart from one additional study visit to coincide with routine medical visits.

We piloted the intervention among eligible English speaking patients enrolled in the second-line clinic between 13 August 2013 and 22 November 2013 with a single recent (within the last 2 months) elevated viral load (≥400 copies/ml). Patients also had to have a cell phone and be willing to receive an SMS message of their choice if they missed a scheduled dose. Intervention cohort patients were identified and recruited from the second-line clinic. An EAMD, otherwise referred to as a Wisepill™ device, was offered to any patient that met the study eligibility criteria and provided informed consent.

The Wisepill™ device is a portable rectangular box (110 mm long × 45 mm wide × 12 mm deep) which holds approximately 30 large or 60 small pills and contains a cell phone SIM card, a 1100 mAH lithium polymer rechargeable battery and a microchip to record time-date stamp within the unit [17]. This wireless device has been previously described [17]. The device monitors and records each time it is opened and assuming that in most cases when the device is opened medication is taken, the data it records can be used as a proxy measure of treatment adherence. In addition, the device can send data to a remote web-based server via cell phone technology and send the patient a reminder text message (SMS) whenever the device has not been opened within a particular time window around the prescribed time for proper adherence. Medical providers can also access online adherence records and graphs. In addition to real-time medication adherence monitoring, a signal (called a “heart beat”) is periodically sent to the device to determine if it is operating properly and to determine battery and signal strength as well as airtime available on the SIM card.

At enrollment (which coincided with intensified adherence counselling) study staff recorded participant and device information so that each participant could be linked to their adherence data and clinical information. Participants returned to the clinic 1 month after enrollment where study staff ensured proper use of the device (e.g., checked battery, signal strength and airtime available). The clinician also reviewed the adherence data collected and had a focused discussion with the participant about their adherence pattern. This additional visit was not part of the usual standard of care that patients receive as part of the second-line clinic. When participants returned for the follow-up viral load test (3–6 months after enrollment) the device was returned and the clinician reviewed the adherence data with the patient. Finally, study staff administered a qualitative interview to determine the feasibility and acceptability of using the device. Questions addressed the participants opinion of using the device, including ease or difficulty of use; potential sigma and loss of confidentiality; and how useful they thought the device was to monitor adherence in real-time.

### Study Variables

For the intervention cohort, the proportion achieving adherence was calculated as the number of “recorded or actual” doses divided by the number of “scheduled (as prescribed)” doses. For this definition, “recorded” doses occurred when the device was opened (morning or evening) within a 3 h window either side of the scheduled time. If it occurred outside of this window period, the dose was defined as “missed”. Where multiple “recorded” doses were recorded in one period (morning or evening), the dose closest to the scheduled dose was considered and all other duplicates were disregarded. Required scheduled dose was calculated as the total number of days multiplied by the number of doses prescribed per day. The total number of days from enrolment was determined as follows: until study staff deactivated the device (definition 1), until the last event recorded (definition 2), or until the repeat viral load date (definition 3). We considered two cut-offs to define good adherence; either taking at least 80 % (missing ≥20 %) or taking at least 95 % (missing ≥5 %) of the prescribed medication [7].

All patient-level data, including information on demographics, medications, laboratory test results and other clinical information were extracted from TherapyEdge-HIV™ prior to the analysis. Viral load testing is not done by the clinic, but by a central lab and therefore those performing the viral load tests were blinded to the study cohorts. Blood samples are sent to the National Health Laboratory Service (NHLS) and viral load and CD4 count results are uploaded directly into TherapyEdge-HIV™ from the NHLS on a daily basis [24]. Because we hypothesized that the device would also encourage patients to remain in care, the primary study outcome was the proportion of subjects in each group alive, in care and virally suppressed by 6 months after eligibility (defined as date of enrollment or adherence counselling for the intervention and comparison cohorts respectively; typically 1–2 months after viral load ≥400 copies/ml on second-line ART). Viral suppression was defined as a repeat viral load <400 copies/ml. The repeat viral load was defined as the first follow-up viral load (VL1) test 3 months after eligibility. We allowed an additional 3 months to capture patients that returned later for their medical visit. The study team did not perform viral load testing and as such could not ensure that all patients had a repeat viral load test. Since retention in care was part of the outcome of interest, this was accounted for in our primary outcome. For the analysis, all patients without a repeat viral load by 6 months were considered failures (i.e., viral load ≥ 400 copies/ml) [19].

In addition, we also estimated cumulative viral suppression by 12 months. For patients who were alive in care, not suppressed by 6 months (VL1), we followed patients for an additional 6 months (VL2) to determine if they could suppress by 12 months after eligibility.

### Statistical Analysis

Continuous variables are described using medians with corresponding interquartile ranges (IQR) and categorical variables are expressed as simple proportions. For the intervention cohort log-binomial regression was used to estimate the association between poor adherence (defined as either ≤ 95 % or ≤ 80 % [7] of required scheduled dose) and unsuppressed virus (≥400 copies/ml) between 3 and 6 months after enrolment.

The effect of the intervention on the primary outcome (alive, in care and virally suppressed 6 months after single elevated viral load on second-line), was estimated using log-binomial regression. As the outcome was relatively common (incidence of ≥10 %), we calculated relative risks and corresponding 95 % confidence intervals (CI). In addition, we conducted a secondary analysis restricted to only patients with a repeat viral load. Models were adjusted for gender, first elevated viral load, time to elevated viral load and CD4 count at first elevated viral load. Loss to follow-up (LTFU) was defined as being ≥3 months late for the last scheduled visit with no subsequent visit. All-cause mortality was ascertained through patient tracing and linkage with the South African National Vital Registration System for patients with a valid South African national identification number (61 %) [24, 25].

### Qualitative Analysis

At the final study visit, study staff administered a qualitative interview consisting of a series of 13 open-ended questions to all study participants (*n* = 49) to assess the feasibility and acceptability of using the EAMD. Participant responses were captured on a paper case report form and the raw data was transcribed and collated. Responses were categorized and coded into key themes, and those with similar content were summarized, with important quotes noted as has been described by Kleiman [26]. Qualitative interviews were read and general patterns or themes were identified. We identified key points to help refine the intervention approach and did not conduct a formal analysis of the qualitative data.

### Cost Per Patient Managed

We collected empirical data on the financial cost of the intervention, incremental to the existing health services, from the provider perspective, using ingredients based methods. Therefore, we included costs of the device and monitoring (e.g., cell phone airtime) for 6 months but excluded staff time. Costs are presented in 2015 US dollars (exchange rate 11.88). We calculated the cost per patient managed assuming that resistance testing costs 300 US dollars per patient and intensified adherence counselling using Wisepill™ EAMD costs 170 US dollars for 6 months. According to the 2015 South African National consolidated guidelines for the management of HIV, adults who have been on a PI containing regimen for at least a year and have not achieved viral suppression would be eligible for resistance testing to determine if third-line is necessary [23]. Cost per patient managed was calculated as the number of patients that did not suppress at 6 months (i.e., would require a resistance test) multiplied by the cost of the EAMD intervention and/or resistance testing divided by the total number of patients in the cohort.

### Compliance with Ethical Standards

All participants in the intervention cohort provided written informed consent. Patient records/information for both comparison cohorts was anonymized and de-identified prior to analysis. Ethics clearance was approved by the University of the Witwatersrand and Boston University ethics committees.

## Results

We enrolled 49 participants in the intervention cohort in addition to 401 patients in the standard adherence cohort and 314 in the intensified adherence cohort. The clinical characteristics of the patients included are presented in Table 1. Patients in the standard adherence cohort and intensified adherence cohort were similar in terms of age at eligibility, gender, first elevated viral load on second-line ART, and time from start of second-line to first elevated viral load. Compared to participants in the intervention cohort, those who were in the standard adherence cohort and intensified adherence cohort differed in terms of gender (more females 70.6 and 72.0 % respectively vs. 59.2 %), had higher median CD4 counts at eligibility (417 and 353 respectively vs. 346 cells/mm^3^), and had a lower median viral load at first elevated viral load on second-line ART (1243 and 1508 respectively vs. 4804 copies/ml).

**Table 1:** Patient demographic and clinical characteristics at eligibility and treatment outcomes for each cohort included at Themba Lethu HIV clinic in Johannesburg, South Africa

Characteristics of patients at the first elevated viral load on second-line ART	Wisepill™ intervention cohort (*n* = 49)	Intensified adherence cohort (*n* = 314)	Standard adherence cohort (*n* = 401)
Gender, female, *n* (%)	29 (59.2 %)	226 (72.0 %)	283 (70.6 %)
Age, years, median (IQR)	37.6 (33.6–45.3)	35.2 (30.5–40.9)	35.2 (30.3–41.2)
CD4 cells/mm^3^, median (IQR)	346 (166–403)	353 (189–558)	417 (260–609)
< 50	1/37 (2.7 %)	11/188 (5.9 %)	16/393 (4.0 %)
51–100	4/37 (10.8 %)	8/188 (4.3 %)	9/393 (2.3 %)
101–250	8/37 (21.6 %)	48/188 (25.5 %)	65/393 (16.5 %)
≥ 250^a^	24/37 (64.9 %)	121/188 (64.4 %)	303/393 (77.1 %)
Time on ART prior to study eligibility (elevated viral load on second-line), months, median (IQR)	48.8 (30.4–68.8)	35.4 (20.1–60.6)	37.6 (21.4–63.5)
Time from start of second-line to study eligibility, months, median (IQR)	11.5 (5.0–23.4)	11.8 (5.1–26.2)	14.8 (6.0–31.2)
Viral load at study eligibility, copies/ml, median (IQR)	4804 (1505–22,455)	1508 (690–15,000)	1243 (648–4461)
Second-line ART regimen, *n* (%)			
ABC_3TC_ LPVr	0	6/314 (1.9 %)	9/401 (2.2 %)
TDF_3TC_ LPVr	17/49 (34.7 %)	74/314 (23.6 %)	143/401 (35.7 %)
AZT_3TC_LPVr	12/49 (24.5 %)	71/314 (22.6 %)	81/401 (20.2 %)
AZT_ddI_LPVr	7/49 (14.3 %)	68/314 (21.7 %)	41/401 (10.2 %)
d4T_3TC_LPVr	13/49 (26.5 %)	95/314 (30.3 %)	127/401 (31.7 %)
Primary outcome at 6 months			
Alive, in care, suppressed, *n* (%)	22/49 (44.9 %)	121/314 (38.5 %)	164/401 (40.9 %)
Alive in care, not suppressed, *n* (%)	26/49 (53.1 %)	170/314 (54.1 %)	232/401 (59.1 %)
Missing viral loads	0	81	101
Not in care	1/49 (0.3 %)	23/314 (7.3 %)	5/401 (1.3 %)
Dead	0	5	1
Loss to follow-up	1	18	4
Final outcome by 12 months			
Alive, in care, suppressed, *n* (%)	33/49 (67.3 %)	218/314 (69.4 %)	312/401 (77.8 %)
Alive in care, not suppressed, *n* (%)	15/49 (30.6 %)	73/314 (23.2 %)	80/401 (20.0 %)
Missing viral load	1	40	20
Not in care	1/49 (0.3 %)	23/314 (7.3 %)	9/401 (2.2 %)
Dead	0	5	1
Loss to follow-up	1	18	8

ABC, abacavir; 3TC lamivudine; LPVr, lopinavir ritonavir; TDF, tenofovir; AZT, zidovudine; ddI, didanosine; d4T, stavudine

^a^Loss to follow-up defined as missing their last scheduled visit ≥3 months

## Evaluation of Adherence Devices Compared to Intensified Adherence Counselling and Standard Adherence Counselling

**Fig. 2:**
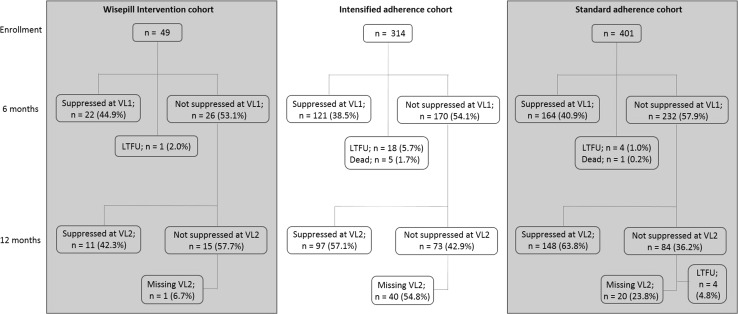
Viral suppression at 6 (VL1) and 12 (VL2) months among patients on second-line ART with an elevated viral load who were enrolled to receive electronic patient adherence monitoring (Wisepill™ Intervention cohort) and comparison cohorts; those receiving intensified adherence counselling or adherence counselling alone (standard adherence cohort). (LTFU; ≥3 months late for the last scheduled visit with no subsequent visit)

By 6 months after the first elevated viral load on second-line ART, 44.9 % (22/49; 95 % CI 31.4–58.9) of participants from the Wisepill™ intervention cohort, 38.5 % (121/314; 95 % CI 33.3–44.0) from the intensified adherence counselling cohort and 40.9 % (164/401; 95 % CI 36.1–45.7) from the standard adherence cohort were alive, in care with a suppressed viral load below 400 copies/ml. At the time of the analysis (close of dataset in November 2014), a total of 67.3, 69.4 and 77.8 % of patients in the intervention, intensified adherence and standard adherence cohort, respectively had re-suppressed their viral load (Fig. 2).

When testing the association between the three groups and our primary outcome we found that patients receiving a combination of EAMD and intensified adherence counselling demonstrated small differences in proportions alive, in care and virally suppressed at 6 months after an elevated viral load on second-line ART when compared to intensified adherence counselling (RR 1.19; 95 % CI 0.85–1.67; risk difference 0.06) or adherence counselling alone (RR 1.12; 95 % CI 0.81–1.56; risk difference 0.04). After adjusting for gender, first elevated viral load, time to elevated viral load and CD4 count at first elevated viral load, we saw no difference in the primary outcome for the different approaches to managing care (Table 2). Viral load suppression was also similar between the comparison cohorts (intensified adherence counselling vs. adherence counselling alone RR 1.04; 95 % CI 0.92–1.17), with a narrower confidence interval. When we restricted the analysis to patients with a repeat viral load, the proportion alive, in care and virally suppressed at 6 months increased from 44.9 to 45.8 % in intervention cohort, from 38.5 to 57.6 % in the intensified adherence cohort and from 40.9 to 55.6 % in the standard adherence counselling cohort. Based on our results, we conducted a sensitivity analysis, and assumed that 50 % of those with a missing viral load (initially assumed they were all failures) were alive, in care and virally suppressed at 6 months. When compared to intensified adherence counselling (RR 0.81; 95 % CI 0.58–1.13) or adherence counselling alone (RR 0.83; 95 % CI 0.60–1.15), the intervention cohort was less likely to achieve the primary outcome, demonstrating the variability in results due to informative missingness of viral load data.

**Table 2:** Association between adherence intervention and being alive, in care and virally suppressed at 6 months

	*n*, %	Alive, in care and virally suppressed	
		Crude RR (95 % CI)	Adjusted RR^a^ (95 % CI)
Full analysis^b^			
Intensified adherence counselling	121/314 (38.5 %)	1.0	1.0
Wisepill™ electronic adherence monitoring	22/49 (44.9 %)	1.19 (0.85–1.67)	1.17 (0.83–1.66)
Standard adherence counselling	164/401 (40.9 %)	1.0	1.0
Wisepill™ electronic adherence monitoring	22/49 (44.9 %)	1.12 (0.81–1.56)	1.06 (0.75–1.49)
Restricted analysis among those with a viral load			
Intensified adherence counselling	121/210 (57.6 %)	1.0	1.0
Wisepill™ electronic adherence monitoring	22/48 (45.8 %)	0.80 (0.57–1.11)	0.79 (0.56–1.10)
Standard adherence counselling	164/295 (55.6 %)	1.0	1.0
Wisepill™ electronic adherence monitoring	22/48 (45.8 %)	0.82 (0.60–1.14)	0.74 (0.53–1.03)

*RR* relative risk, *CI* confidence interval

^a^Model adjusted for gender, first elevated viral load, time to elevated viral load and CD4 count at first elevated viral load

^b^For the full analysis, all patients without a repeat viral load by 6 months were considered failures (i.e. viral load ≥400 copies/ml)

### Cost Analysis

If we consider that 61.5 % of the total intensified adherence cohort (*n* = 314) would require resistance testing after 6 months then the cost per patient managed would be 184 US dollars (193 × 300 US dollars ÷ 314). If one implemented the EAMD intervention then theoretically the proportion requiring resistance testing would drop from 61.5 to 55.1 % (see Table 2—difference in the proportion alive, in care and virally suppressed at 6 months) but the cost per patient managed would increase to 335 US dollars (173 × 470 US dollars [$300 for resistance testing +$170 for Wisepill monitoring] plus 141 × 170 US dollars ÷ 314).

### Intervention Participants and Adherence Levels

Intervention cohort participants used the devices for a median of 4.9 months (IQR 4.2–5.1). The majority (85.7 %) were on a standard second-line regimen of lamivudine (3TC) and LPVr with various non-nucleoside reverse transcriptase inhibitors (stavudine 26.5 %; tenofovir 34.7 %; zidovudine 38.8 %) at study enrolment. The majority (92 %; *n* = 45) of participants took ART twice daily while the remaining 8 % took a once daily regimen. The median time on ART was 6.1 years (IQR 3.7–7.7) at enrolment into the study. Median cumulative adherence among intervention participants was 90.7 % (IQR 79.2–96.2), 85.4 % (IQR 68.7–96.8) and 86.7 % (IQR 68.6–95.4) for definition 1, 2 and 3 respectively. We describe the study population, stratified by adherence calculated from enrolment until last event recorded (definition 2) (Table 3). Overall, 63 % of all participants enrolled in the study (59 % of females and 70 % of males) took more than 80 % while only 27 % (13/49) took more than 95 % of their prescribed medication. The proportion of patients taking >80 % of doses dropped from 76 % at 1 month post-enrolment to 53 and 49 % at 2 and 3 months respectively.

**Table 3:** Characteristics of patients on second-line ART with an elevated viral load and enrolled to receive electronic patient adherence monitoring, stratified by adherence calculated from enrolment until last event recorded (definition 2) (*n* = 49)

Characteristic	Adherence > 80 % (*n* = 31)	Adherence ≤ 80 % (*n* = 18)
Gender, female, *n* (%)	17/29 (58.6 %)	12/29 (41.4 %)
Age, years, median (IQR)	42.9 (36.2–50.7)	39.2 (38.1–44.5)
Education—secondary level and beyond, *n* (%)	21/35 (60.0 %)	14/35 (40.0 %)
Employed, *n* (%)	21/35 (60.0 %)	14/35 (40.0 %)
First-line ART regimen, *n* (%)		
d4T_3TC_EFV	24/36 (66.7 %)	12/36 (33.3 %)
TDF_3TC_EFV	2/4 (50.0 %)	2/4 (50.0 %)
AZT_3TC_EFV	2/3 (66.7 %)	1/3 (33.3 %)
Other	3/6 (9.6 %)	3/6 (50.0 %)
Time on first-line ART, median (IQR)	6.2 (3.4–7.8)	5.7 (3.7–7.4)
Second-line ART regimen, *n* (%)		
TDF_3TC _LPVr	11/17 (64.7 %)	6/17 (35.3 %)
AZT_3TC_LPVr	10/12 (83.3 %)	2/12 (16.7 %)
AZT_ddI _LPVr	4/7 (57.1 %)	3/7 (42.9 %)
d4T_3TC_LPVr	6/13 (46.2 %)	7/13 (53.8 %)
Time from second-line initiation to eligibility (i.e. elevated viral load), median months (IQR)	9.8 (4.1–21.8)	18.4 (6.2–30.5)
CD4 cells/mm^3^ at eligibility, median (IQR)	367 (173–568)	373 (168–413)
<250	8/12 (66.7 %)	4/12 (33.3 %)
≥250	14/24 (58.3 %)	10/24 (41.7 %)
Missing	9/13 (69.2 %)	4/13 (30.8 %)
Follow up viral load (3–6 months after eligibility)	280 (100–781)	601 (198–1500)
<400 copies/ml	16/22 (72.7 %)	6/22 (27.3 %)
400–1000 copies/ml	9/14 (64.3 %)	5/14 (35.7 %)
1000–10,000 copies/ml	5/9 (55.6 %)	4/9 (44.4 %)
≥10,000 copies/ml	1/3 (33.3 %)	2/3 (77.7 %)
Missing	0/1 (0 %)	1/1 (100.0 %)

ABC, abacavir; 3TC, lamivudine; LPVr, lopinavir ritonavir; TDF, tenofovir; AZT, zidovudine; ddI, didanosine; d4T, stavudine

By the date of dataset closure (November 2014; allowing a minimal of 12 months of follow-up), 48 (98 %) patients had a repeat viral load while one patient was lost to follow-up and had no repeat viral load. Of those with a repeat viral load, 46 % (95 % CI 32–60; 22/48) were undetectable (<400 copies/ml) while 29 % (95 % CI 18–43; 14/48) were between 400 and 1000 copies/ml and 25 % (95 % CI 14–39; 12/48) were greater than 1000 copies/ml. For the primary outcome analysis we considered the one lost patient to be a failure as they did not have a repeat viral load.

Compared to those with good adherence (>80 %), participants with poor adherence (≤80 %) had a higher risk for a follow up viral load ≥400 copies/ml [relative risk (RR) 1.47 95 % CI 0.97–2.23]. Using different definitions of adherence (Table 4), patients with poor adherence were more likely to have a repeat viral load ≥400 copies/ml, although the estimate lacked precision due to our small sample size.

**Table 4:** Association between poor adherence (≤80 or 95 %) and a repeat viral load ≥400 copies/ml in the intervention group

	Viral load ≥ 400 copies/ml *n* %	Crude RR (95 % CI)	Adjusted RR^a^ (95 % CI)
*Definition 1*			
Adherence > 80 %	17/35 (48.6 %)	1.0	1.0
Adherence ≤ 80 %	10/14 (71.4 %)	1.47 (0.91–2.37)	1.47 (0.97–2.23)
Adherence > 95 %	8/14 (57.1 %)	1.0	1.0
Adherence ≤ 95 %	19/35 (54.3 %)	0.95 (0.55–1.64)	1.06 (0.63–1.78)
*Definition 2*			
Adherence > 80 %	15/31 (48.4 %)	1.0	1.0
Adherence ≤ 80 %	12/18 (66.7 %)	1.38 (0.85–2.25)	1.35 (0.86–2.12)
Adherence > 95 %	7/13 (53.9 %)	1.0	1.0
Adherence ≤ 95 %	20/36 (55.6 %)	1.03 (0.58–1.85)	1.15 (0.66–2.0)
*Definition 3*			
Adherence > 80 %	16/33 (48.5 %)	1.0	1.0
Adherence ≤ 80 %	11/16 (68.8 %)	1.42 (0.88–2.30)	1.48 (0.98–2.24)
Adherence > 95 %	7/13 (53.9 %)	1.0	1.0
Adherence ≤ 95 %	20/36 (55.6 %)	1.03 (0.58–1.85)	1.15 (0.66–2.0)

^a^Model adjusted for time to elevated viral load calculated from start of second-line ART until first elevated viral load. The total number of days was determined as follows: from enrolment until study staff deactivated the device (definition 1), from enrolment to last event recorded (definition 2), or until the repeat viral load date (definition 3)

Among the 49 intervention cohort subjects, 10,332 events (i.e., device openings) were recorded. No participant’s device had a signal at any point indicative that it could not pick up a signal or communicate with the server. A small number (13.1 %) of the drug intakes had a signal strength below 10 (but any signal strength value is regarded as adequate) and 10.8 % of all drug intakes had a battery strength less than 3700 mV suggesting that the battery was low and needed recharging. Drug intakes were however still recorded even when the battery voltage dropped to the lowest at 3400 mV.

### Acceptability

We report good uptake of the device with only two patients who were offered it refusing to participate. Overall, 96 % of participants reported that Wisepill™ helped them to remember to take their medication. Most patients did not have a problem telling people what the device was when asked, one participant said he just told people it was a “machine that helps me remember to take my medication”. Participants reported that the device was “easy to carry around”, “it’s portable”, “I can keep all my tablets in one place”, “it reminds me to take my medication on time”, “looks like a modern day gadget”, “better than carrying packets of pills” and “it is a good box”. Participants felt that the device not only reminded them to take their pills when they forgot but it helped them to be more careful and responsible about taking their pills because they knew that someone was “watching” or monitoring them. On the negative side participants reported that “it would send reminders even when I took my medication”, “I didn’t like that it had to be charged”, “it is too big”, “people asked about the box” and “timing of SMS was inconvenient”.

Participants suggested that the device should be combined with a reminder (e.g., alarm, buzzer etc.), preferably in one device. Participants also reported that there should not be long delays between forgetting to take their medication and receiving a SMS reminder. Some patients also complained about getting multiple reminders despite actually taking their medication. Participants also mentioned that the device could be smaller and that it should be designed to fit into trouser pockets for convenience, especially in men.

Overall, participants were optimistic about the device and their responses indicated that they found the device “acceptable and useful”. One participant reported that “It was great to be reminded to take my medication” while another participant mentioned that “This box is very precious, it is a nice box. I don’t have to be reminded by my wife. I wish I can keep it”.

## Discussion

We conducted a pilot study in Johannesburg, South Africa to determine if short-term use of electronic patient adherence monitoring devices can improve adherence in patients failing second-line antiretroviral therapy. By 6 months after eligibility, 44.9 % (95 % CI 31.4–58.9) of participants in the intervention cohort re-suppressed their virus without a regimen switch. This is similar to what has been reported for re-suppression on first-line ART in South Africa (40 %) [27]. A significant proportion of patients had virologic failure despite good adherence, highlighting those most likely to benefit from resistance testing and third-line drugs.

Despite the fact that participants found the device “acceptable and useful”, Wisepill™ resulted in modest improvement in the primary outcome over intensified adherence counselling. We demonstrate that compared to those who received intensified adherence counselling, patients receiving combination intervention of electronic adherence monitoring and intensified adherence counselling were slightly more likely to be alive, in care and virally suppressed at 6 months after an elevated viral load on second-line ART (44.9 vs. 38.5 %), for an additional cost of 151 US dollars per patient managed. In order for the intervention to be considered, either the effectiveness would have to be improved (>95 % alive, in care and virally suppressed) or the cost of the intervention would have to be dramatically reduced (<18 US dollars). We could not demonstrate that EAMD could significantly improve long-term adherence, as measured by viral suppression, but the results are encouraging since patients that re-suppress early are more likely to remain virally suppressed [28]. Our findings are similar to those recently reported from a randomized controlled trial among patients on first-line ART [19]. Viral suppression may not be the most appropriate way to measure the effectiveness of EAMD and other outcomes such as treatment interruptions, missed clinic visits and long term viral suppression may be more suitable.

Overall re-suppression rates were low but expected given the study population. A recent study among patients experiencing treatment failure on protease-inhibitor based second-line ART showed that 46 % (51/111) of patients reach re-suppression at first follow-up viral load after an adherence support intervention similar to the intensified adherence cohort described here [28]. Others have also reported that that 46 % of patients experiencing failure on second-line ART achieve viral re-suppression within 3 months, 18 % within 6 months and 5 % within 9 months of a second-line failure intervention lasting 3 months [29]. The encouraging news is that patients that re-suppress early (e.g., at the first follow-up viral load) are three times more likely to remain virally suppressed, compared to those that reach viral suppression later. An additional 41 % of patients reach viral re-suppression at the second or third follow-up viral load test [28]. This suggests that longer follow-up, beyond 6 months, may be needed in order to accurately determine the impact of adherence interventions.

Virological failure may be caused by a number of factors, including drug resistance, length of time on treatment and poor adherence [28, 30]. We demonstrate that participants with poor adherence (≤80 %) had a higher risk for a follow-up viral load ≥400 copies/ml (71.4 vs. 48.6 %). In a study by Shuter and colleagues, 80 % of LPVr recipients achieved an undetectable viral load (<400 copies/ml) despite a mean adherence rate of 73 % and substantial ART experience [31, 32]. LPVr’s forgiveness of non-adherence is likely attributable to two separate factors; first is its pharmacokinetic profile, which exceeds 50 % effective concentration for more than 24 h in normal volunteers, so patients without resistant virus can miss one of two daily doses but still maintain therapeutic levels of the drug between doses and, second, is the low frequency at which the virus develops resistance to this agent [32].

We also report that adherence dropped at 2 and 3 months post-enrolment. Recent evidence from this cohort has revealed social and behavioural factors including use of herbal/traditional medicine, alcohol and depression were significantly associated with failure to re-suppress viral load on second-line ART following intensified adherence counselling (results not shown). Therefore, interventions to address these barriers should be considered as part of the adherence intervention package to improve adherence over time [33, 34].

### Limitations

Our findings should be considered in light of the study limitations. Firstly, compared to those in the Wisepill™ intervention cohort, the intensified adherence cohort and standard adherence cohort had a considerable amount of missing viral load data (25.8 and 25.2 % by 6 months and 12.7 and 5.0 % by 12 months). Patients with missing viral loads were considered failures [19], potentially leading to an overestimation of the effect of the Wisepill™ intervention. To address this we included a sensitivity analysis to determine the potential impact if a proportion of those with missing viral loads (50 %) had suppressed their viral load.

Second, we could not confirm adequate cellular signal at the participants’ home or work prior to enrolment in the study [17]. We did not have approval to contact participants (e.g., telephonically or through home visits) if there was a lapse in signal and could therefore not determine if the cause was technical failure (e.g., low battery power or low airtime) or behavioural (e.g., opting not to use the device). However, to minimize this during the study, participants were sent an SMS reminder to recharge the battery when low battery power was detected. Other factors including signal strength and issues with cell phones (e.g., lost, left at home etc.) could have resulted in more patients being classified as poorly adherent. Better cell-phone coverage would reduce delays in transmissions, possibly improving the effectiveness of the intervention.

Third, this study was not a randomized controlled trial aimed at measuring the effect of the intervention but rather a pilot study to generate preliminary estimates of effectiveness, generate preliminary cost data and evaluate the feasibility of EAMD. The pre-post design may result in secular trends or periodic variation. Study enrolment took place near the holiday period which may have resulted in a longer duration between counselling and the repeat viral load for some. To overcome this we used the first repeat viral load within 6 months of eligibility. Furthermore, some participants travelled out of South Africa and cell phone coverage did not include international roaming. These patients would have recorded missed drug scheduled doses in their adherence pattern and also would not have received SMS reminders when low battery was detected.

Participation in a study may in itself motivate adherence. Patient motivation can be enhanced by free accessible care, approachable and supportive healthcare workers, broad social acceptance of ART, and past first-hand experiences with AIDS-related co-morbidity and mortality [35]. Although we did not conduct a formal analysis of the qualitative data, EAMD have been shown to be feasible in similar settings [36]. Although EAMD allows for monitoring and optimizing adherence in real-time (e.g., with the SMS reminders), we limited patient interaction to clinic visits, when it may have already be too late for some patients to prevent failure or drug resistance. Also, important to note is that opening the device does not necessarily translate into participants taking the drugs correctly, and this intervention is dependent on the patient using the device correctly. These are important factors that should be considered when implementing adherence reminder devices in routine settings, emphasising the importance of context, examining specific features of the intervention and rigorous evaluation of implementation impact and performance [14, 15].

Last, we restricted the intervention period to 3 months. It may be important to consider a longer intervention period (±6–9 months) for patients on second-line ART as adopted in other studies [37, 38]. Murphy and colleagues also report that time to virologic suppression is most rapid among patients with 91–100 % adherence, so those with ≤80 % adherence may take longer to reach virologic suppression [38].

## Conclusion

Adherence strategies increase the durability of second-line ART, decrease the need for costly third-line regimens and prevent unnecessary genotyping tests [28]. Our findings are important since few studies have investigated adherence interventions among patients failing second-line ART. In addition, this is one of the first studies to investigate the use of EAMD in a second-line ART population, the population where it could be cost-effective at current prices. Results are encouraging especially since patients that re-suppress at first follow-up viral load are more likely to remain virally suppressed [28]. Finally, adherence is associated with virological outcomes; a mere 10 % improvement in the rate of adherence has been shown to double the chance of achieving an undetectable viral load and reduce the risk of virological failure by as much as 73 % [4, 38].
